# Efficacy of Gatifloxacin in Acute Bacterial Corneal ulcer

**DOI:** 10.12669/pjms.296.4175

**Published:** 2013

**Authors:** Sameen Afzal Junejo, Arshad Ali Lodhi, Munawar Ahmed, Mahesh Kumar, Mustafa Kamal, Murtaza

**Affiliations:** 1 Sameen Afzal Junejo, FCPS, Liaquat University of Medical and Health Sciences Jamshoro, Sindh, Pakistan.; 2 Arshad Ali Lodhi, FCPS, Liaquat University of Medical and Health Sciences Jamshoro, Sindh, Pakistan.; 3 Munawar Ahmed, FCPS, Liaquat University of Medical and Health Sciences Jamshoro, Sindh, Pakistan.; 4 Mahesh Kumar, DCP (Path), Liaquat University of Medical and Health Sciences Jamshoro, Sindh, Pakistan.; 5 Mustafa Kamal, MBBS, Liaquat University of Medical and Health Sciences Jamshoro, Sindh, Pakistan.; 6 Murtaza, MBBS, Liaquat University of Medical and Health Sciences Jamshoro, Sindh, Pakistan.

**Keywords:** Bacterial Corneal ulcer, Gatifloxacin, gram-negative bacteria, Gram-positive bacteria, Therapeutic action

## Abstract

***Objective*** **:** To assess the efficacy of Gatifloxacin 0.3% ophthalmic solution in infective corneal ulcer.

***Methods*** *: This *observational (non comparative) clinical analysis was done at the Department of ophthalmology unit-II, Liaquat University Eye hospital Hyderabad of Liaquat University of Medical and Health Sciences Jamshoro / Sindh, Pakistan from April 2010 to March 2012. All the subjects who fulfilled the inclusion criteria were registered. Anterior segment examination was performed. Corneal staining and sensitivity test was done to exclude viral and paralytic element. Corneal samples were collected for gram’s staining and culture sensitivity tests. After getting the preliminary laboratory results, Gatifloxacin 0.3% ophthalmic solution was used in bacterial corneal ulcer every 30 minutes for first twenty four hours, and every one hour till three days. On obtaining better response the drops were used every two hours up to 7 days. The treatment was continued with tapering of dosage for three weeks. After total recovery (re-epithelialization of corneal epithelium) the drops were used two times a day for one more week.

***Results*** **:** The total of 170 patients (male=68.8%; female=31.2%) were recruited. Culture sensitivity examination revealed staphylococcus (36.5%), followed by fungi (24.1%). Pseudomonas thus detected were 10%. Gatifloxacin showed highest sensitivity and lowest resistance i.e. 87.65% and 12.35% respectively against gram positive and gram negative isolates.

***Conclusion: ***Gatifloxacin 0.3% ophthalmic solution due to its strong activity against various gram-positive and gram-negative microbes is strongly effective in the treatment of acute bacterial keratitis.

## INTRODUCTION

Infectious keratitis is no doubt a major and growing problem in the developing countries. It sometimes becomes sight-threatening and results in permanent visual loss due to delayed diagnosis and inappropriate treatment.

Acute bacterial keratitis once developed is rapidly progressive. Initially corneal ulcer is formed with surrounding corneal epithelium and stromal edema. The surrounding swollen area undergoes necrosis. The inflammatory products like cytokines cause migration of inflammatory cells in anterior chamber resulting to severe reaction and development of hypopyon.^1^ In severe cases thinning of corneal stroma leads to perforation. Healing of the corneal ulcer may give rise to corneal opacity, irregular astigmatism and decreased visual acuity.^1^

The risk factors are ocular injury, wearing of contact lenses, ocular surface disorders, dry eyes, corneal sensational loss etc. Staphylococci, Streptococci, Pseudomonas and Enterobacteriaceae are the most pathogenic bacteria to cause bacterial keratitis.^2^

Before the invention of fluoroquinolones, fortified antibiotics used to be the drugs of choice in the treatment of bacterial keratitis. With the passage of time fortified antibiotics were replaced with broad spectrum fluoroquinolone antibiotics that had proved themselves quite successful in preventing and treating ophthalmic infections. The second generation topical ciprofloxacin 0.3% solution was used as an alternative against many resistant strains.^3^ Shortly afterwards ciprofloxacin resistance against gram negative and coagulase-negative Staphylococcus species in bacterial keratitis became known.^4^ The third-generation fluoroquinolone, levofloxacin did not show any superiority over second generation ciprofloxacin against bacterial keratitis.^5^ Fourth generation Gatifloxacin 0.3% ophthalmic solution alone replaced second and third generation fluoroquinolone and has been considered as most effective in killing the bacteria responsible for infective keratitis.^6^

The purpose of this study was to review the current medical management options available for treating the microbial keratitis.

## METHODS

This observational clinical analysis was done at the tertiary eye care center for twenty four months between April 2010 and March 2012. All the registered patients were examined at outpatient department.

***Inclusion Criteria:*** The individuals of eight years of age and older (male and female) presenting for the first time with active corneal infection of one week history without perforation with no history of any previous medication and surgical intervention were registered for the study.

***Exclusion Criteria: ***The patients with viral dendritic corneal ulcers and neurotropic keratitis (painless corneal ulcers), showing Known hypersensitivity to gatifloxacin and other quinolones, exposed to general practitioners and quacks, not completing post treatment follow up of four weeks were not included.

The subjects with bacterial keratitis were enquired for rapid onset of pain, photophobia, and decreased vision. History was taken regarding ocular trauma, contact lens wear, and topical use of steroids, diabetes mellitus, acute or chronic dacryocystitis and any systemic illness that would have caused the corneal infection. The verbal / written consent of patients was obtained and examination performed as follows;

The visual acuity was recorded on Snellen chart and E-chart. During examination of anterior segment on slit lamp bio-microscope, cornea was examined for epithelial ulceration (shape, size in mm, depth and margins) with and without suppuration. The anterior chamber was examined to exclude anterior chamber reaction with or without hypopyon. The culture guided approach consisted of collecting samples for microbiological and culture sensitivity examination from area of infected cornea by following method; 

In adults the cornea was anesthetized with topical instillation of local anesthetic (proparacaine hydrochloride), and corneal scrapping was obtained with a sterile No.15 surgical scalpel blade from the base and edges of the corneal affected area. While in children general anesthesia was induced. A portion of each scrapping was examined microscopically for the presence of bacteria, fungi or acanthamoeba by using Gram staining, 100g/L potassium hydroxide (KOH) and Giemsa staining methods respectively. In few cases the slides treated with KOH were also treated with Lactophenol Blue for the identification of fungi. In selected patients Zeil-Neilson stain was also used. 

Another portion was inoculated onblood agar, chocolate agar, Mac-Conkey agar, Sabouraud's agar, brain heart infusion broth respectively, in C-shaped streaks and cultured for the potential growth of, bacteria, fungi or acanthamoeba. Sabouraud's agar slants were incubated at 28^º^C mixed with gentamicin 100umg/ml to suppress bacterial growth while others at 37^º^C. Most of the time the growth results were obtained within first 24 hours. In case of no response the process was extended to two weeks for slow growing bacteria or fungi. Isolated bacteria were tested by chemical reaction for identification. The susceptibility of gram +ve and gram –ve bacteria against the listed antibiotics was noted and classified as 3-plus (most sensitive), 2-plus (moderate), 1-plus (mild sensitive) and negative (resistant). The resistance to antibiotics was evaluated with the standard disc diffusion method according to the modified test recommended by the NCCLS. 

All the tests were performed by the same microbiologist/ clinical pathologist.

The diagnosis was established after confirming one of the following;

1. Corneal scraping of the material revealed bacterial and fungal presence in smear.
2. The same pathogens grew in two culture media.

Immediately after obtaining the microscopic results the subjects were started with frequent instillation of Gatifloxacin ophthalmic drops as monotherapy and some cases added with tobramycin as combination. Topical treatment was added with oral paracetamol as analgesic.

The treatment was changed when laboratory results recommended another antibiotic more sensitive than the initially started. The patients were examined carefully during the course of treatment. After satisfactory improvement the subjects were discharged with request to complete post treatment follow up of four weeks.

## RESULTS

The total of one hundred and seventy eyes of 170 patients with suspected acute microbial keratitis presented for the first time and completed post treatment follow up criteria of four weeks were registered for this study. The characteristics of the subjects are mentioned in Table-I.

**Table-I:** Characteristics of Patients with Acute Microbial Keratitis: (n=170).

*Sex*	* N/O Patients*	*Percentage % *
Male	117	68.8
Female	53	31.2
*Age Years:*		
08	22	12.9
Up to 20	47	27.7
Up to 40	86	50.6
Over 40	15	08.8
*Residency:*		
Rural	111	65.2
Urban	59	34.8
*Socio Economic Status:*		
Upper	27	15.8
Middle	43	25.2
Lower	100	59.0

**Table-II:** Source of Acute Microbial Keratitis: n=170

*Source*	*N/O Patients*	*Percentage %*
Ocular surface disease	71	41.7
Adnexial disease	42	24.7
Contact Lens wear	16	09.4
Agriculture trauma	41	24.2

**Table-III:** Culture Sensitivity of Isolated Microorganisms: n = 170

*Isolates*	*N/O Eyes*	*Percentage %*
*Gram Positive Cocci*		
Staphylococcus Aureus	62	36.5
Staphylococcus Epidermidis	24	14.1
Streptococcus Pneumoniae	15	08.8
*Gram Negative Bacilli*		
Pseudomonas	17	10.0
Proteus	07	04.1
Fungi	41	24.1
* Mixed Microbs*	*04*	*02.4*
*Total*	170	100%

**Table-IV:** Antibiotic sensitivity and resistance (n=170).

*Antibiotics*	*Sensitivity*		*Resistance*	
	*Frequency*	*%*	*Frequency*	*%*
Tobramycin	119	70.00%	51	30.00%
Ofloxacin	134	78.82%	36	21.18%
Ciprofloxacin	125	73.53%	45	26.47%
Fucidic acid	91	53.53%	79	46.47%
Gentamycin	115	67.65%	55	32.35%
Gatifloxacin	149	87.65%	21	12.35%
Chlorophenicol	115	67.65%	55	32.35%
Neomycin	72	42.35%	98	57.65%
Norfloxacin	123	72.35%	47	27.65%

**Table-V:** Visual Acuity Record: n=170

*Before Treatment*	*N/O Patients*	*V/A with %*
Better	27	6/12 better 15.9%
Moderate	47	6/36 better 27.6%
Poor	61	6/60 FC 35.9%
Not Recorded	35	------------ 20.6%
*After Treatment*		
Better	48	DO 28.2%
Moderate	70	DO 41.1%
Poor	17	DO 10.0%

V/A=Visual Acuity; DO=Same as above; FC=Counting Finger

**Fig.1:**
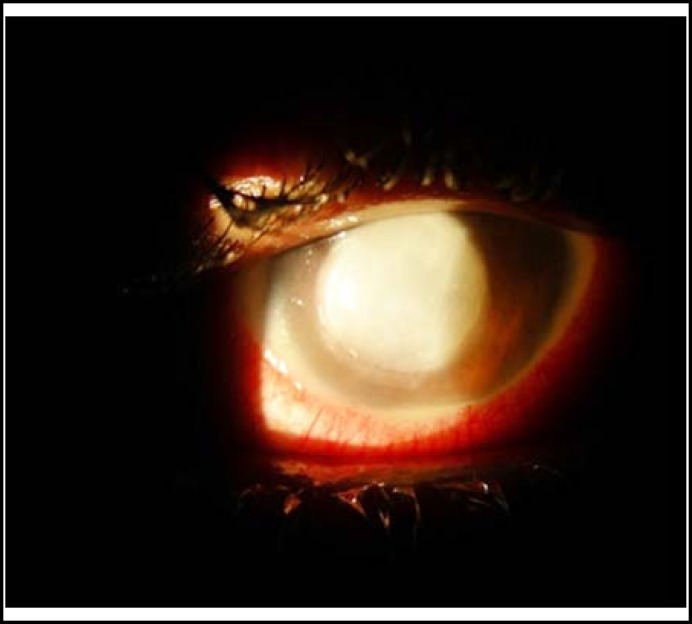
A 30 year old lady with large central corneal abscess presented at Liaquat University Eye Hospital

**Fig.2:**
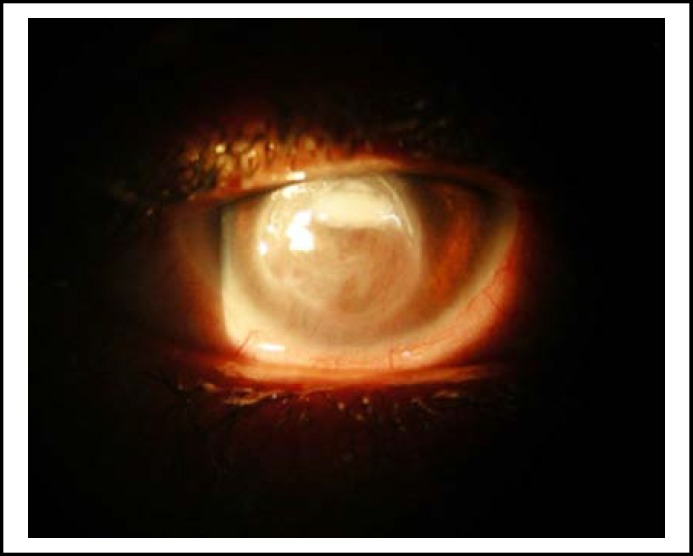
A 30 year old lady with central corneal abscess improved rapidly. Photograph taken after 7 days of treatment

**Fig.3:**
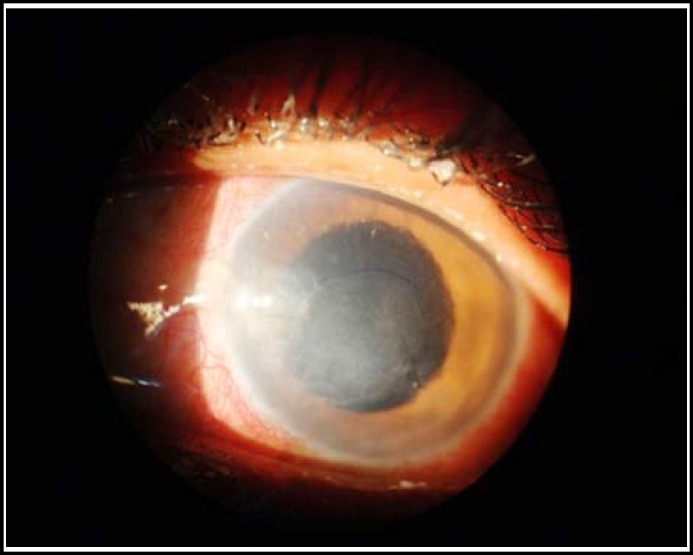
A 30 year old lady with right corneal abscess after complete healing. Photograph taken one month after treatment

Table-II states the source of acute microbial corneal ulcer. Most of the subjects developed corneal infections due to ocular surface disorders i.e. 41.7% followed by adnexal diseases i.e. 24.7%.(Fig.1). The material collected by corneal scraping was sent to the laboratory for microbiology tests. Among positive isolates, staphylococcus aureus was the most commonly detected organism in 52 (30.5%) eyes as shown in Table-III.

After performing antibiotic sensitivity test against both gram positive and gram negative isolates it was confirmed that the Gatifloxacin fourth generation fluoroquinolone proved to be highly effective (87.65%) and superior to the rest of other fluoroquinolones used in the sensitivity tests as mentioned in Table-IV.

The visual acuity was critical due to corneal infections at first visit but during the course of treatment the patients started showing recovery, there was a remarkable improvement (Fig. 2 and 3) in visual acuity particularly at the end of follow up as mentioned in Table-V.

## DISCUSSION

Bacterial keratitis is a potentially blinding ocular condition which involves the cornea at any age from infancy to adult hood and can result to irreversible loss of vision if not managed at early stages. Appropriate treatment could minimize the incidence of corneal damage.^7^^,^^8^

Males are affected more than the females, supported by different studies from different regions of Pakistan.^9^ The statement is consistent to the studies from India as well.^10^ In this study also the males were affected in majority perhaps due to traditional outdoor/field occupation.

In one study from southern Pakistan the culture sensitivity examinations of corneal scrapings revealed gram positive cocci with preponderance of Staphylococcus aureus.^11^ In our work up the isolated staphylococcus aureus were 36.5%, staphylococcus pneumonae 8.8% and pseudomonas 10.0% which simulates with this study and another study from Pakistan showing majority of Staphylococcus Aureus as the majority causative organism.^12^

In our study and other studies from Pakistan discussed earlier the most frequently isolated Gram negative Bacillus was Pseudomonas aeruginosa. Rapidly progressive nature of bacterial keratitis, demands early intensive treatment to prevent blinding complications. Majority of cases with bacterial keratitis show rapid response with early treatment and do not require culture sensitivity and other microbiological investigations. Corneal scrapings are indicated in patients with large centrally located deep corneal ulcers not responding to combination of broad spectrum antibiotic treatment.^13^ In this study the outcome of early treatment with intensive topical antibiotic instillation was quite rewarding. Newer emerging antibiotics have revolutionized the treatment of microbial keratitis.^14^

Among the fluoroquinolone group of anti-microbial agents, gatifloxacin obtained by the slight modification of previous generation fluoroquinolones side chain is getting increasingly prescribed. The broad spectrum gatifloxacin is capable of inducing potent anti-bacterial activity as compared to the prior quinolone ophthalmic antibiotic compositions.^15^^,^^16^

In this study fourth generation fluoroquinolone gatifloxacin has been proved ideal in combating with both gram positive and gram negative microbes due to its capability of low bacterial resistance.

Shah VM et al.^17^ in 2010, Sueke H et al.^18^ in 2010, Chawla B et al.^19^ in 2010 and Bharathi MJ et al.^20^ in 2010 in their recent work compared the effect of topical gatifloxacin 0.3% ophthalmic solution with ciprofloxacin 0.3%, a second-generation fluoroquinolone, in the treatment of bacterial keratitis. The microbiological analysis revealed that gatifloxacin strongly acted against gram positive and gram negative bacteria (92.9%) compared to ciprofloxacin.^17^^-^^20^

This study also correlates with rest of above mentioned studies where gatifloxacin showed 87.65% activity on record. In this study the bacterial isolates showed low resistance against gatifloxacin which correlates with the study conducted by Constantinou et al. from Australia in 2007, which states the capability of fluoroquinolones due to their structural modification and dual inhibition mechanism.^21^

In the current study, the outcome of visual acuity of the subjects was also good during the last days of follow up because of early diagnosis and appropriate selection of antibiotics. Gatifloxacin should be empirically used in the treatment of acute bacterial keratitis because of its rapid action and deep penetration.^22^ Due to paucity of advanced updated microbiological facilities we could not explore our work in detail. There is definitely a need for further extended research on this issue.

## CONCLUSION

The fourth generation fluoroquinolones are effective against both gram-positive and gram-negative isolates. Gatifloxacin has great value and exhibited better anti-microbial activity as compared to the other fluoroquinolones in the treatment of acute microbial keratitis.

## Authors Contribution:

AA and MA: Contributed for drafting, methodology, patients selection, and literature search.

MK: Helped in performing micro biological examinations and data collection.

MK and M: Contributed in follow up of patients and maintaining their record etc.

SAJ: Critical review of the manuscript and approval of the final version to be published.

## Notes

***Source of funding:*** Self-funded.
